# Supplementary material for: Macrophage differentiation is marked by increased abundance of the mRNA 3’ end processing machinery, altered poly(A) site usage, and sensitivity to the level of CstF64
Source: Front Immunol. 2023 Jan 25;14:1091403. doi: 10.3389/fimmu.2023.1091403 (PMC9905730; doi:10.3389/fimmu.2023.1091403)
Supplement: Supplementary file 1 [file DataSheet_1.pdf]

Supplementary Material

1 Supplementary Figures and Tables

1.1 Supplementary Tables

Supplementary Table S1. Primers and shRNA sequence used in this study.

Purpose	Primer name	Forward primer 5’-3’	Reverse primer 5’-3’
RT-qPCR for gene expression	CD86 total mRNA	GACTGAGTAACATTCTCTTTGTGATGGCC	CTACTAGCTCACTCAGGCTTTGG
	ICAMI total mRNA	CCAATGTGCTATTCAAACTGC	CAGCGTAGGGTAAGGTTCTTG
	JUN total mRNA	AAGTTGCGACGGAGAGAAAA	ACGCAACCCAGTCCAACTT
	FOS total mRNA	GACCGAGCCCTTTGATGAC	GCCACTGTGCAGAGGCTC
	NFKB1 total mRNA	CAAGCAGCTCTGCAGCAG	ACTGTCATAGATGGCGTCTG
	IKZFI total mRNA	CACGTGATGGACCAAGC	CGGCTTGTGCAGCTGGTAC
	OSM total mRNA	CTCCTGGACCCCTATATACG	GTGTGGCATTGAGGGTCTG
	IL12A total mRNA	GAAAGTCCTGCCGCGCC	GTTTCTGGCCAAACTGAGGTG
	GAPDH mRNA	TGATGACATCAAGAAGGTGGTGAAG	TCCTTGGAGGCCATGTGGGCCAT
	ACTB mRNA	CATGTACGTTGCTATCCAGGC	CTCCTTAATGTCACGCACGAT
Processing activity	MYC Total	ATTACAGGTGTGAGCCAGGG	AGCCTGCCTCTTTTCCACA
	MYC Span	ATCATTGAGCCAAATCTTAAGTTGTG	CTCTGAAGGGGCAATTGATGA
	ACTB Total	TCAAGGTGGGTGTCTTTCCT	CCTGCTTGCTGATCCACATC
	ACTB Span	GCTTTTGGTCTCCCTGGGA	CTGCACTCTGGGTAAGGACA
CstF64 overexpression (For PCR cloning)		CTTACGGCTAGCGCCACCATGGCGGGTTTGAC (Nhe I enzyme site)	CATTAGACCGGTAGGTGCTCCAGTGG (Ase I enzyme site)

Purpose	Sequence
CstF64 knockdown shRNA #1	CGTAGAGAACGATCCACCG
CstF64 knockdown shRNA #2	TGGCATTCCAACCTGAGCC
CstF64 knockdown shRNA #3	CGTGCATCTATTCCTCGGG
Oligo d(T) adapter for 3’RACE	GCGAGCACAGAATTAATACGACTCACTATAGGTTTTTTTTTTTTTTVN
Reverse anchor-primer for 3’RACE	GCGAGCACAGAATTAATACGACT
SERPINB1 3’RACE exon Fwd	GGCCTGAAGAAGATTGAGGAAC
PTCH1 3’RACE exon Fwd	CAGCACCTTGTTACAGACAAGAAG
PSAT1 3’RACE exon Fwd	CATAGGTCTGTGGGAGGC
MRPL44 3’RACE exon Fwd	GCACTTTTCATCAGGGACTTCTTAATTAC
CIAPIN1 3’RACE exon Fwd	GTGGAAACTGCTACCTGGGC
EIF1 3’RACE exon Fwd	GCGTTTAAGAAAAAGTTTGCCTG

Supplementary Table S2. Antibodies used in this study.

Catalogue no.	Name	Host	MW
SC-20052	CD16 Or FCGR3B	Mouse monoclonal	50 kDa
A6554	CD68	Rabbit polyclonal	110-140 kDa
SC-55593	HLA-DRα	Mouse monoclonal	36 kDa
SC-18853	ICAM1 (6.5B5)	Mouse monoclonal	85-110 kDa
SC-374650	CD38 (H-11)	Mouse monoclonal	45 kDa
SC -58951	CD14 (5A3B11B5)	Mouse monoclonal	53-55 kDa
ab8227 (Abcam)	Beta Actin	Rabbit polyclonal	43 kDa
SC-365062	GAPDH (G-9)	Mouse monoclonal	37 kDa
Bethyl A301-357A	CFI_m68 or CPSF6	Rabbit	68 kDa
Bethyl	CFI_m25 or NUDT21	Rabbit	25 kDa
SC-393880	CFI_m59 Or CPSF7	Mouse monoclonal	52 kDa
SC-398392	FIP1L1 (C-10)	Mouse monoclonal	67 kDa
SC-166281	CPSF160 or CPSF1 (G-10)	Mouse monoclonal	160 kDa
SC-165983	CPSF100 or CPSF2 (A-11)	Mouse monoclonal	100 kDa
SC-374466	WDR33 (D-1)	Mouse monoclonal	146 kDa
SC-393001	CPSF73 or CPSF3 (C-3)	Mouse monoclonal	73 kDa
SC-393316	CPSF30 or CPSF4 (D-1)	Mouse monoclonal	30 kDa
Bethyl A301-250A	CSTF50 or CSTF1	Rabbit polyclonal	50 kDa
SC-376575	CstF-77 (G-5) or CSTF3	Mouse monoclonal	77 kDa
SC-398862	CstF64 (H-1) or CSTF2	Mouse monoclonal	64 kDa
Bethyl A301-487A	CSTF2T/TauCSTF64/CstF64τ	Rabbit polyclonal	70 kDa
Bethyl AAS02642C	hClp1	Rabbit polyclonal	41 kDa
Bethyl A303-706A	Pcf11	Rabbit Polyclonal	173 kDa
SC-135410	Symplekin (H308)	Rabbit polyclonal	150 kDa
SC-32915	PAP (H-300)	Rabbit polyclonal	64 kDa (may be 90 kDa)

1.2 Supplementary Figures

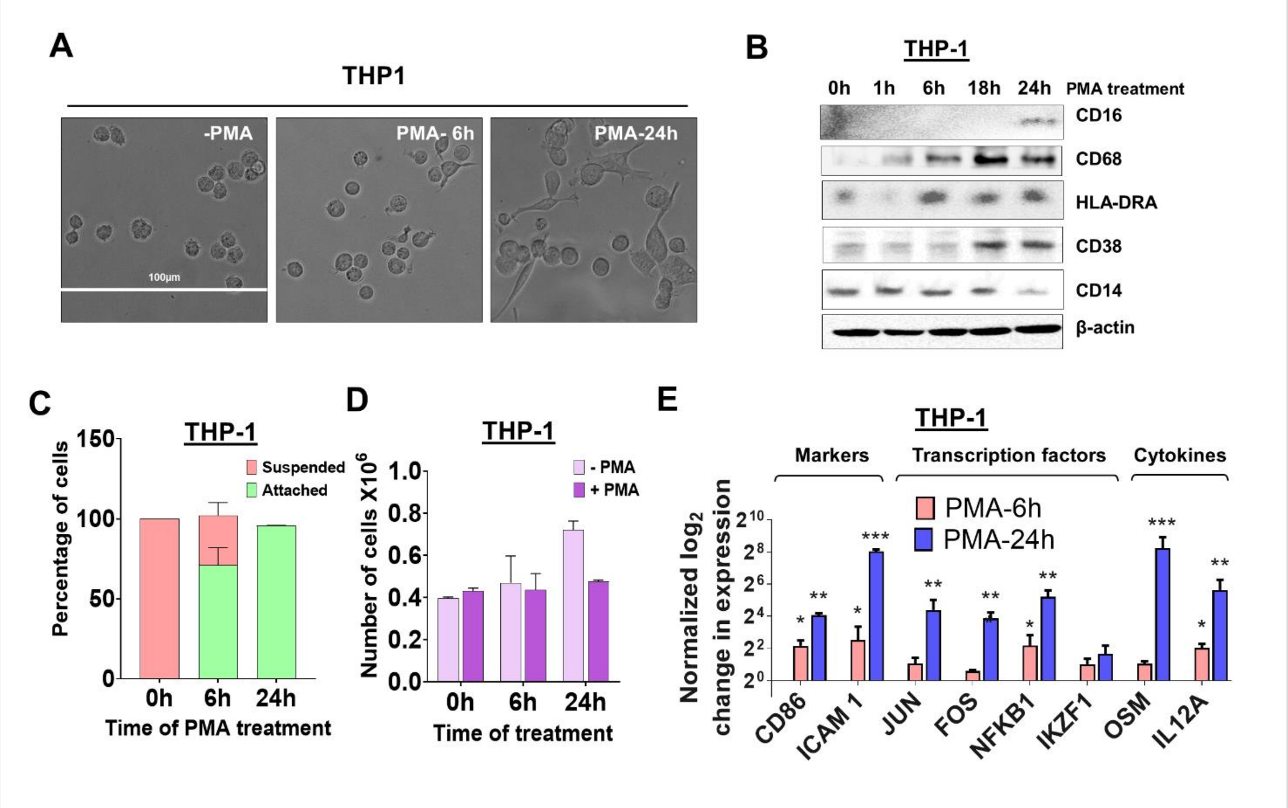

Supplementary Figure S1. Morphological changes and altered expression of cell-surface markers and mRNAs during differentiation of THP-1 cells and human primary monocytes.

(A) The effect of differentiation on THP-1 cellular morphology. THP-1 cells exposed to PMA (30 nM) for 6 and 24 hours and observed by phase contrast microscopy (40X). (B) Western blots of total cell lysates from THP-1 cells differentiated with PMA (30 nM) for 0, 1, 6, 18 and 24 hours. Blots were probed with antibodies against CD16, CD68, HLA-DRA, ICAM1, CD38 and CD14 with β-actin as the loading control. (C) Attachment assay for THP-1 cells, performed as described for Fig. 1C. (D) Proliferation assay for THP-1 cells performed as described for Fig. 1D. (E) Effect of THP-1 differentiation on the expression level of mRNAs from genes involved in macrophage differentiation and function. RT-qPCR assays performed for genes as described in Fig. 1E. The figure represents mean ± SE from three independent experiments. P value <0.05 was considered significant, where * = $P \leq 0.05$; ** = $P \leq 0.01$; *** = $P \leq 0.001$.

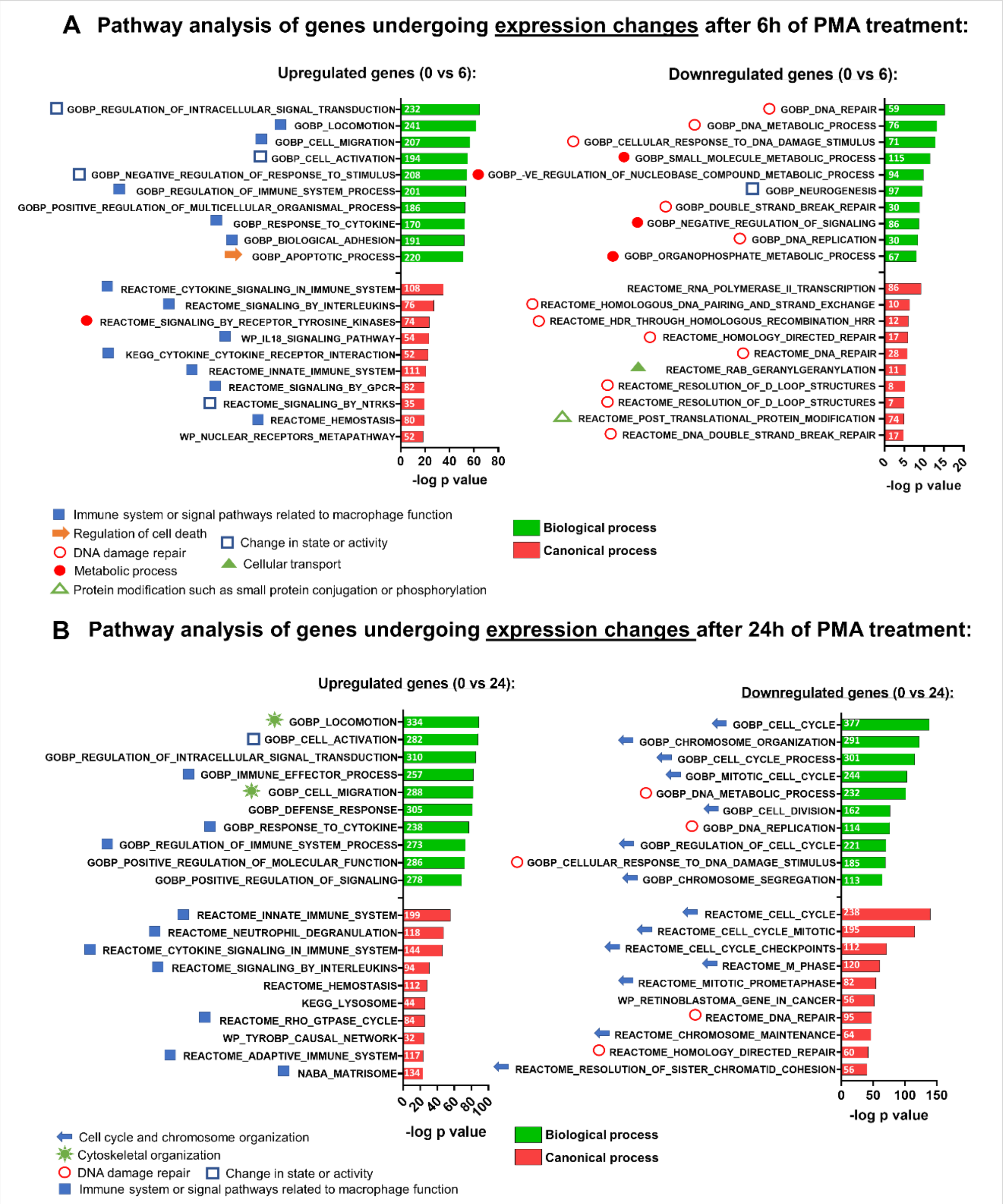

Supplementary Figure S2. Gene set enrichment analysis of mRNAs with expression changes in U937 cells after PMA treatment.

Functional annotation clustering of mRNAs whose expression is regulated after 6h (A) and 24h (B) of differentiation. The 10 most significant GO-process enriched gene groups according to GSEA analysis are ranked based on negative $\log_{10}$ (P-values) for upregulated (left panel) and downregulated (right panel) transcripts. Green indicates biological process; red, canonical process. Pathways classified according to specific cellular processes are grouped together as indicated by the individual symbols at the bottom of the graphs.

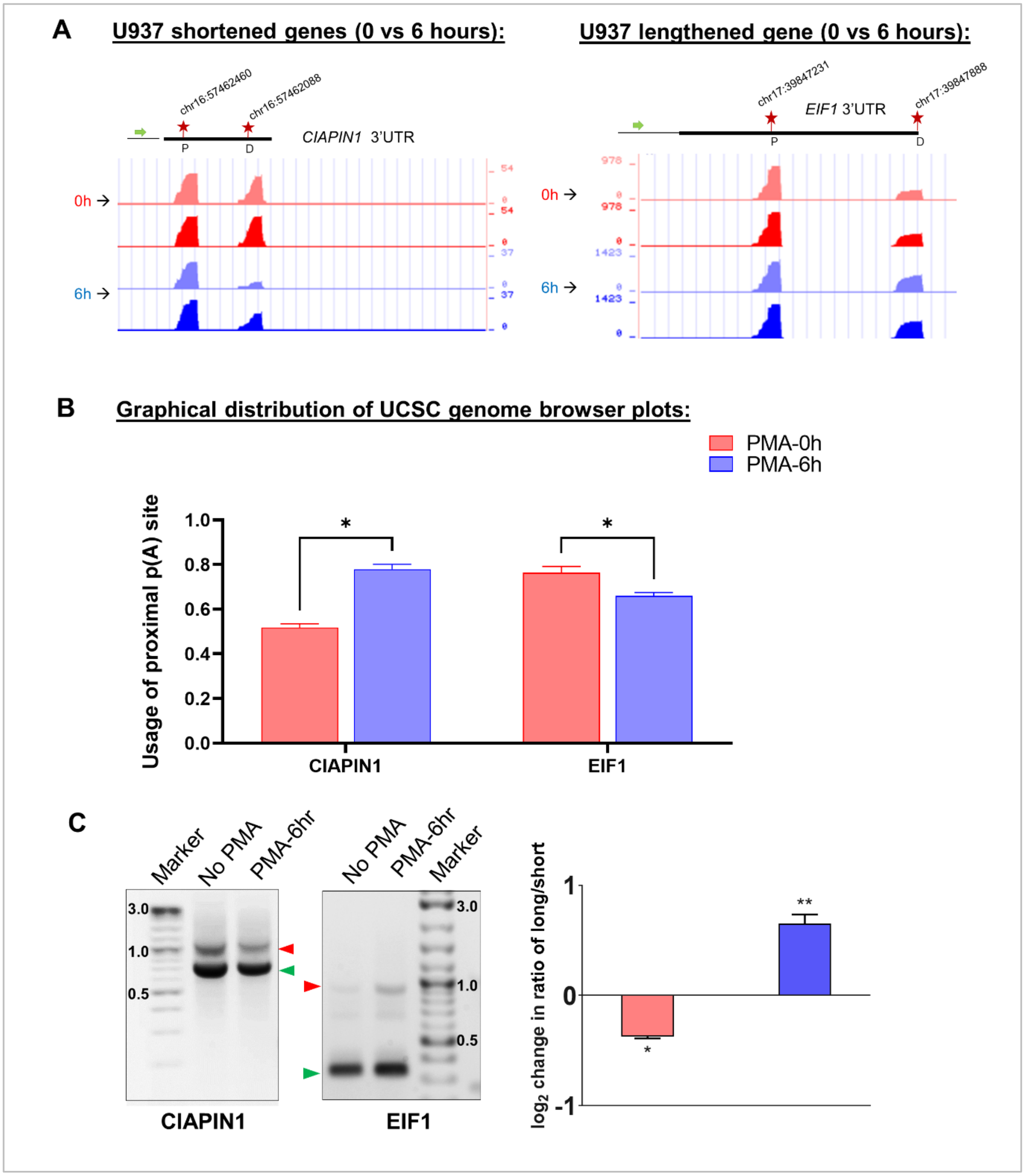

Supplementary Figure S3. Changes in poly(A) site use in U937 cells after 6 hours of PMA treatment and validation by 3'RACE.

(A) UCSC genome browser plots of RNA sequencing tracks highlighting the 3′-UTR profile differences for shortened and lengthened genes after 6h PMA treatment with respect to control (0h). The colors of the tracks represent 0h (red) and 6h (blue). Proximal (P) and distal (D) poly(A) sites are indicated with red stars. The green arrow defines the direction of the coding strand, blue arrow defines the direction of chromosome co-ordinates and tag counts are indicated on the y axis. Additionally, positions and chromosome co-ordinates of the annotated PACs are indicated at the top of each browser plot. (B) Quantitative bar graphs reflecting the differences in APA for shortened and lengthened targets. The mean relative usage of proximal poly(A) site with respect to the total read

counts at the proximal and distal poly(A) sites as visualized in the UCSC genome browser is plotted for each target. Unpaired t-test was performed to determine the significance between the treatment groups and P value <0.05 was considered significant where * = $P \leq 0.05$. (C) Representative 1% agarose gel images for the 3′ RACE RT-PCR from U937 cells (untreated and 6h PMA-treated) for shortened gene CIAPIN1 and lengthened gene EIF1 (upper panel) using gene specific primer at the last exon-exon junction and reverse anchor primer. Normalized intensities of long and short bands for each PCR were quantified and a long/short ratio determined and normalized to the No PMA control. The graph (lower panel) represents mean ± SE from at least two independent experiments. P value <0.05 was considered significant, where * = $P \leq 0.05$; ** = $P \leq 0.01$.

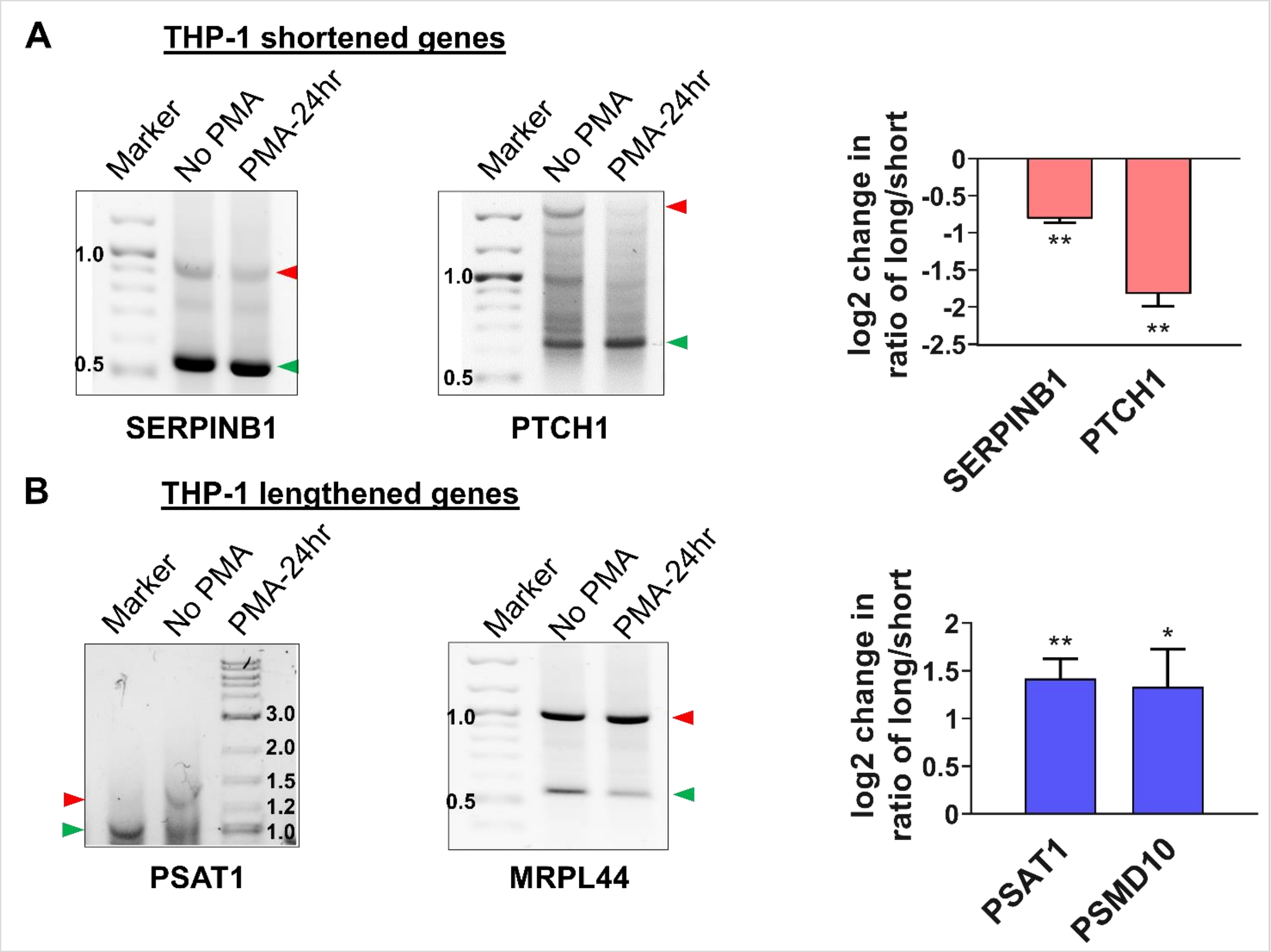

Supplementary Figure S4. Validation of APA events in THP-1 and human primary monocytes by 3' RACE.

Representative 1% agarose gel images for the 3′ RACE RT-PCR from control and differentiated THP-1 cells and human primary monocytes for shortened genes SERPINB1 and PTCH1 (A and C), lengthened genes PSAT1 and PSMD10 (B and D) with gene specific primer at the last exon-exon junction and reverse anchor primer. Normalized intensities of long and short bands for each PCR were quantified and a long/short ratio determined and normalized to the No PMA or undifferentiated controls. The corresponding graphs (right) represents mean ± SE from at least two independent experiments. P value <0.05 was considered significant, where * = $P \leq 0.05$; ** = $P \leq 0.01$.

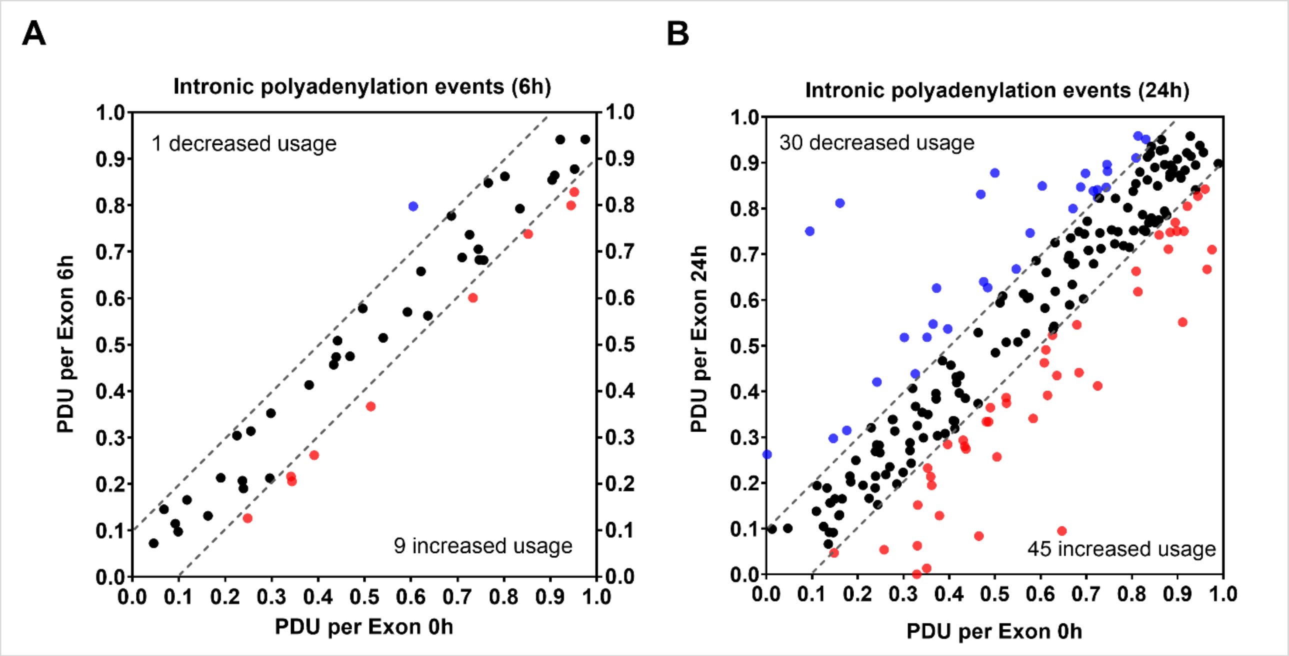

Supplementary Figure S5. Evidence of intronic polyadenylation events.

(A) and (B) mRNAs undergoing intronic polyadenylation in U937 cells treated with PMA for 6h and 24h, classified according to their delta PDUI changes in both directions. Every exon with multiple PACs having "splicing APA" event (Supplementary Tables S3 and S4) is plotted here, and the PDU for 0h is on the x-axis and 24h on the y-axis. Depending upon the significance levels reported by the DPAC pipeline, blue dots indicate lengthening or decreased usage of the poly(A) site whereas pink dots indicate shortening or increased usage of the poly(A) site. Dotted grey lines indicate a change in PDU of 0.1 in either direction and the events (black dots) within the lines are considered not significantly altered.

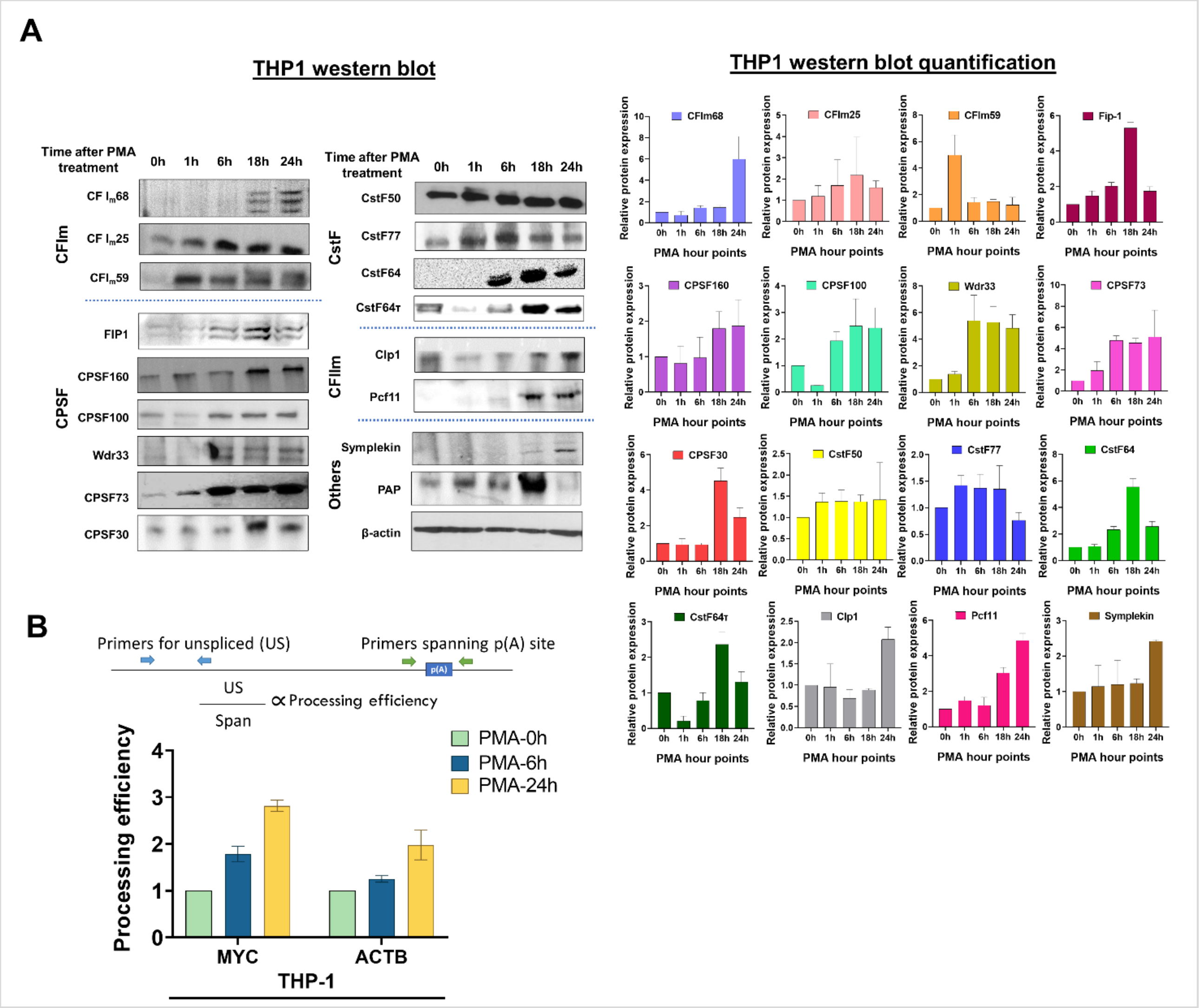

Supplementary Figure S6. Effect of macrophage differentiation on the expression of C/P proteins and mRNAs in THP-1 cells and human primary monocytes.

(A) Whole cell lysates from THP-1 cells treated with PMA for 0, 1, 6, 18 and 24 hours were separated by 10% SDS-PAGE and western blotting was performed for the indicated subunits of the C/P complex, with β-actin as the loading control. Each western blot (left panel) was performed in three biological replicates of the differentiation process and the quantified data is shown in the right panel. (B) Processing efficiency of MYC and ACTB transcripts for THP-1 cells. The bar graph shows the ratio of unspliced (US) RNA transcripts (detected by RT-qPCR with a primer pair upstream of the poly(A) site) to unprocessed RNA (detected by RT-qPCR with a primer pair that spans the poly(A) site) as shown in the schematic. The cDNA preparation was done by using random hexamers.

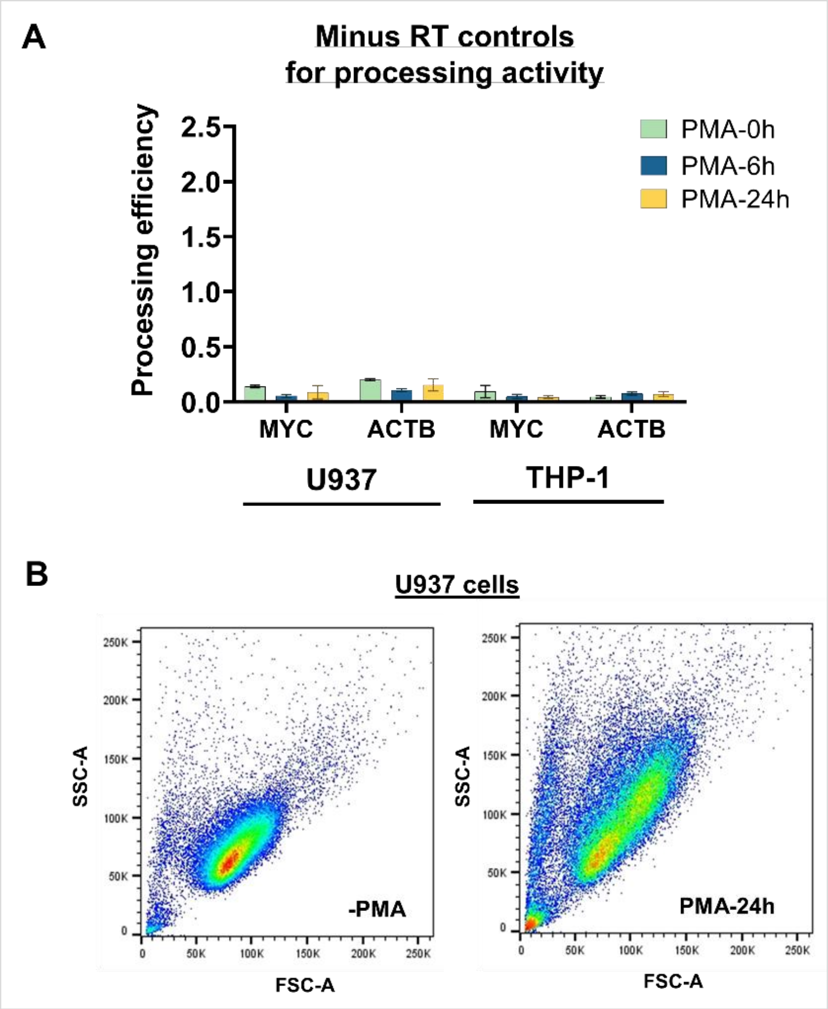

Supplementary Figure S7. Processing efficiency minus RT control and cellular complexity of U937 cells by FACS.

(A) Minus RT control for processing efficiency of MYC and ACTB transcripts for U937 and THP-1 cells. PCR was done by adding equivalent amount of RNA that was used to prepare cDNA for Fig. 6D and S6D (1µg). The bar graph shows the ratio of unspliced (US) RNA transcripts (detected by RT-qPCR with a primer pair upstream of the poly(A) site) to unprocessed RNA (detected by RT-qPCR with a primer pair that spans the poly(A) site) as shown in Fig. 6D. (B) Changes in cellular complexity determined by FACs analysis of side (SSC) and forward scattering (FSC) values for untreated and 24h PMA-treated U937 cells.

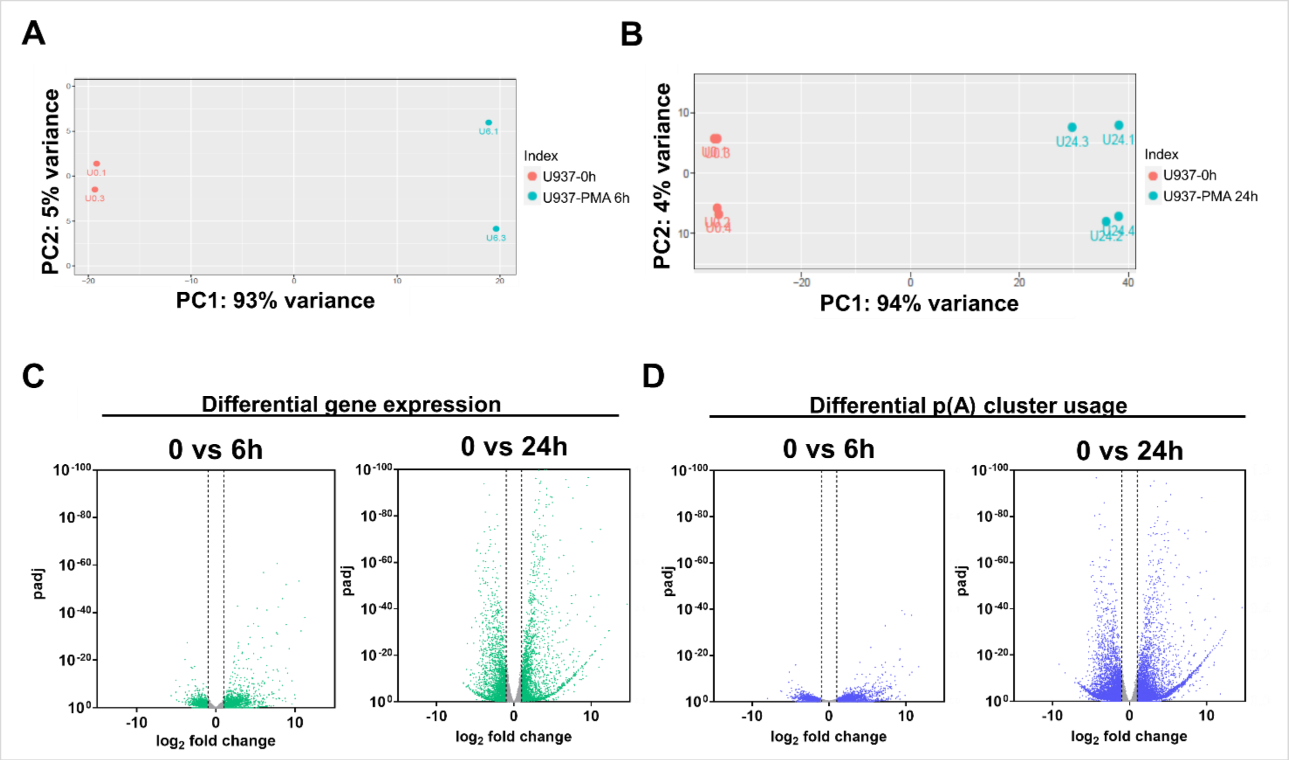

Supplementary Figure S8. Analysis of p(A) site mapping data.

(A and B) Principal component analysis (PCA) plot of U937 Poly(A)-Click-sequencing data from samples of the control (0h) and cells treated with PMA for 6 hours (A) or 24 hours (B). Each dot represents a sample and each color represents the time of PMA treatment. (C and D) Volcano plots illustrating changes in gene expression counts and PAC counts. They were generated by plotting the $\log_2$ fold change values vs p_{Adj} value for 6 hour (left panel) and 24 hour (right panel) PMA-treated U937 cells with respect to control undifferentiated ones for differential gene expression counts (C) and poly(A)-cluster counts (D). The gene counts for 6h and 24h collapse all mapped p(A) sites within one gene into one number and provides differential gene expression data, and PACs are defined as groupings of p(A) sites that are found within 25 nucleotides of one another. The gene counts in each PAC are used to determine relative changes in site selection between samples. Differential Gene or PAC count is defined as >1.5 fold change with p_{Adj} <0.1 (independent hypothesis weighting).
